# Sexual- und Verhütungsverhalten von Jugendlichen und jungen Erwachsenen in Deutschland. Aktuelle Ergebnisse der Repräsentativbefragung „Jugendsexualität“

**DOI:** 10.1007/s00103-021-03426-6

**Published:** 2021-10-01

**Authors:** Sara Scharmanski, Angelika Heßling

**Affiliations:** Abteilung S – Sexualaufklärung, Verhütung und Familienplanung, Referat S3 – Aufgabenkoordinierung, Nationale und internationale Zusammenarbeit, Forschung und Fortbildung, Bundeszentrale für gesundheitliche Aufklärung (BZgA), Maarweg 149–161, 50825 Köln, Deutschland

**Keywords:** Repräsentativbefragung, Reproduktive Gesundheit, Sexualaufklärung, Kontrazeption, Hormonelle Verhütung, Representative survey, Reproductive health, Sexual education, Contraception behaviour, Hormonal contraception

## Abstract

**Hintergrund:**

Seit 1980 führt die Bundeszentrale für gesundheitliche Aufklärung (BZgA) in regelmäßigen Abständen die Repräsentativbefragungen „Jugendsexualität“ durch. Dieses kontinuierliche Monitoring generiert Erkenntnisse zur sexuellen und reproduktiven Gesundheit von jungen Menschen in Deutschland, die eine wichtige Basis einer bedarfs- und zielgruppengerechten Entwicklung von Maßnahmen der Sexualaufklärung und Familienplanung darstellen.

**Ziel:**

Das aktuelle Sexual- und Verhütungsverhalten von Jugendlichen und jungen Erwachsenen soll anhand erster deskriptiver Ergebnisse der 9. Trendwelle zusammenfassend dargestellt werden.

**Material und Methoden:**

An der Befragung nahmen insgesamt *N* = 6032 Jugendliche und junge Erwachsene teil. Die Datenerhebung erfolgte in 2019 als kombiniert mündlich-schriftliche Interviews (Computer-assisted Personal Interviewing, CAPI).

**Ergebnisse:**

Ein zentraler Befund der vorliegenden Trendwelle ist, dass der Anteil an Jugendlichen, die beim ersten Geschlechtsverkehr jünger als 17 Jahre sind, seit einigen Jahren rückläufig ist. Zur Kontrazeption setzten Jugendliche am häufigsten das Kondom ein, die Nutzung der Pille ist im Trend deutlich rückläufig.

**Diskussion:**

Die Daten der aktuellen Trendwelle weisen ein sicheres und verantwortungsbewusstes Verhütungsverhalten von jungen Menschen in Deutschland nach. Trotzdem gilt es, das Engagement im Bereich der sexuellen Gesundheitsförderung aufrechtzuerhalten und zielgruppenspezifische Präventionsmaßnahmen weiter auszubauen. Denn nur so kann die sexuelle und reproduktive Gesundheit der nachfolgenden Generation gewährleistet werden.

## Abstract (en)

**Background:**

Since 1980, the Federal Centre for Health Education (BZgA) has regularly conducted the “Youth Sexuality” representative surveys. This continuous monitoring generates insights on the sexual and reproductive health of young people in Germany. The survey provides an important basis for the development of sexuality education and family planning measures.

**Aim:**

The current sexual and contraceptive behavior of adolescents and young adults will be summarized using initial descriptive results from the ninth iteration of the survey.

**Methods:**

A total of *N* = 6032 adolescents and young adults participated in the survey. Data collection was conducted by computer-assisted personal interviewing (CAPI) in 2019.

**Results:**

A key finding of this iteration is that with regards to the age of the first sexual intercourse, the proportion of adolescents younger than 17 years has been declining for several years. For contraception, adolescents most frequently used condoms, and use of the pill has decreased.

**Discussion:**

The data from the current iteration indicate safe and responsible contraceptive behavior among young people in Germany. Nevertheless, it is important to maintain the commitment in the field of sexual health promotion and expand prevention measures for specific target groups. This is the only way to ensure the sexual and reproductive health of the next generation.

## Hintergrund

Die Bundeszentrale für gesundheitliche Aufklärung (BZgA) ist durch das Schwangerschaftskonfliktgesetz (SchKG) beauftragt, Informationen zur Sexualaufklärung und Verhütung zu entwickeln und bundesweit kostenfrei zur Verfügung zu stellen [1].

Zur Erfüllung dieses gesetzlichen Auftrages entwickelt die BZgA altersadäquate Medien, wie etwa die Website loveline.de, hält zielgruppenspezifische Informationsmaterialen, wie beispielsweise Aufklärungsbroschüren, vor und fördert Maßnahmen und Forschung im Bereich der sexuellen und reproduktiven Gesundheit. Dabei ist die Orientierung an dem „Public Health Action Cycle“ (PHAC; dt.: gesundheitspolitischer Aktionszyklus) zentral, der gesundheitsbezogene Interventionen und gesundheitspolitisches Handeln strukturiert und systematisiert [2]. Die Orientierung am PHAC setzt jedoch eine fundierte Datenbasis sowie ein kontinuierliches Monitoring der Zielgruppenbedarfe und Implementierungsbedingungen voraus. Denn nur durch gesicherte Evidenzen können zielgruppenspezifische Bedarfe identifiziert, Ansprachen zielgruppengenau ausgerichtet, die Effektivität überprüft und notwendige strategische und operative Anpassungen vorgenommen werden.

Vor diesem Hintergrund haben die Durchführung und Förderung von großen Surveys zu Themen der sexuellen und reproduktiven Gesundheit in der BZgA eine lange Tradition [3–6]. Ein bedeutendes Monitoring ist in diesem Zusammenhang die repräsentative Querschnittsbefragung zur Jugendsexualität, die seit 1980 regelmäßig durchgeführt wird [7, 8]. Anhand dieser Studiendaten können Informationen über das jeweils aktuelle Verhalten und die aktuell vorhandenen Einstellungsmuster von Jugendlichen und jungen Erwachsenen zu Themen der Sexualaufklärung und Kontrazeption generiert werden.

Im vorliegenden Beitrag werden erste Ergebnisse der 9. Trendwelle der Jugendsexualitätsstudie vorgestellt. Im Fokus stehen hier vor allem der Einstieg ins aktive Sexualleben, Partnerschaften und Beziehungen sowie das Verhütungsverhalten Jugendlicher von 14–17 Jahren und junger Erwachsener von 18–25 Jahren in Deutschland.

## Methoden

Seit knapp 40 Jahren wird die vorliegende Querschnittsbefragung regelmäßig repliziert, wobei das methodische Grundgerüst weitgehend unverändert blieb.

Die Stichprobenanlage hingegen musste im Laufe der vergangenen knapp 40 Jahre aufgrund sich verändernder gesellschaftlicher Rahmenbedingungen immer wieder angepasst werden (z. B. nach der Wiedervereinigung in den 1990er-Jahren, zunehmende Migration ab den 2000er-Jahren). Seit 2014 werden ergänzend zu Jugendlichen auch Teilstichproben von jungen Erwachsenen zwischen 18 und 25 Jahren als Zielgruppen der Jugendsexualitätsstudie befragt.

Im Folgenden werden das methodische Vorgehen, die Stichproben und das eingesetzte Erhebungsinstrument sowie die statistischen Analysen der 9. Welle der Jugendsexualitätsstudie beschrieben.

## Vorgehen

Die Datenerhebung der 9. Welle erfolgte im Zeitraum von Mai bis Oktober 2019 von der Kantar GmbH mit der CAPI-Methode (Computer-assisted Personal Interviewing) als kombiniert mündlich-schriftliche Interviews. Der Mantelfragebogen wurde im persönlichen Face-to-Face-Interview erhoben, intimere Fragen – wie beispielweise Fragen nach Masturbation – füllten die Jugendlichen und jungen Erwachsenen am Laptop aus (Selbstausfüllerteil).

Die Befragung fand in der häuslichen Umgebung der Jugendlichen bzw. jungen Erwachsenen und meistens ohne Anwesenheit Dritter statt. Im Falle von Minderjährigen waren die Eltern im Haushalt während der Befragung anwesend. So konnte gewährleistet werden, dass wenn die Jugendlichen nach der Befragung vertiefende Informationen über Sexualität und Verhütung wünschten, ihnen theoretisch Ansprechpersonen zur Verfügung standen.

Sowohl die Erziehungsberechtigten als auch die Jugendlichen bzw. jungen Erwachsenen wurden im Vorfeld umfassend über Ziel und Zweck der Studie sowie die Datenverarbeitung schriftlich und mündlich aufklärt; die Befragung erfolgte nur nach Einwilligung der Eltern und Jugendlichen bzw. jungen Erwachsenen.

Eine intensive Schulung vor Durchführung der Befragung und die langjährige Expertise des Feldinstitutes in diesem Forschungsbereich stellten sicher, dass die Interviewerinnen und Interviewer die Befragung altersangemessen, kultursensibel und empathisch durchführen konnten.

## Stichprobe

Inhaltlich ist neben den Aspekten der Sexualaufklärung v. a. das Thema Verhütung und Familienplanung zentral. Vor diesem Hintergrund wurde schon 1980 ein disproportionaler Stichprobenansatz in Bezug zur Geschlechterverteilung gewählt und mehr Mädchen als Jungen befragt. Durch dieses Vorgehen ist gewährleistet, dass auch im Falle von differenziellen Analysen einzelner Subgruppen hinreichend große Fallzahlen zur Verfügung stehen. Eine anschließende Gewichtung der Datensätze gleicht die Disproportionalität bei der Stichprobenziehung aus, sodass die Repräsentativität der Ergebnisse für die Grundgesamtheit erhalten bleibt (vgl. „Statistische Analysen“).

In der vorliegenden 9. Welle der Jugendsexualitätsstudie wurden gemäß Stichprobenanlage 8 disproportionale Teilstichproben realisiert, die sich jeweils aus der Kombination der drei Hauptkriterien Geschlecht (weiblich vs. männlich)^1^, Altersgruppe (14 bis 17 vs. 18 bis 25 Jahre) und kulturelle Herkunft (mit vs. ohne Migrationshintergrund) ergeben. An der Befragung nahmen *N* = 2024 Mädchen und *N* = 1532 Jungen zwischen 14 und 17 Jahren sowie *N* = 1580 junge Frauen bzw. *N* = 896 junge Männer zwischen 18 und 25 Jahren teil. Weitere ungewichtete Merkmale der Teilstichproben sind in Tab. 1 dargestellt.

**Table 1:** 

Teilstichprobe	Alter 14–17 Jahre			Alter 18–25 Jahre			Gesamt		
	–	*n*	%	–	*n*	%	–	*n*	%
Geschlecht	Weiblich Männlich	2024 1532	57 43	Weiblich Männlich	1580 896	64 36	Weiblich Männlich	3604 2428	60 40
Migrationshintergrund	Ja Nein	1034 2522	29 71	Ja Nein	840 1636	34 66	Ja Nein	1874 4158	31 69
Bildungsniveau	Niedrig Mittel Hoch	422 1575 1500	12 45 43	Niedrig Mittel Hoch	316 728 1407	13 30 57	Niedrig Mittel Hoch	738 2303 2907	12 39 49

Das Bildungsniveau der Befragten wurde über die besuchte Schule und/oder den angestrebten bzw. vorhandenen Schulabschluss operationalisiert. Ein Migrationshintergrund wurde aufgenommen, wenn ein Jugendlicher oder ein junger Erwachsener selber oder mindestens ein Elternteil nicht mit deutscher Staatsbürgerschaft geboren wurde [9].

Die Auswahl der Zielpersonen erfolgte nichtrandomisiert nach dem Quota-Verfahren [10], wobei die Quoten unterschiedlichen Veröffentlichungen des Statistischen Bundesamtes^2^ entnommen sind [11–13]. Merkmale der Quotierung waren Geschlecht, Alter, Wohnregion, kulturelle Herkunft und Bildungsniveau bzw. besuchte Schulform/vorhandener Schulabschluss.

Zur Gewährleistung einer adäquaten regionalen Verteilung diente der Standort der interviewenden Personen, der relativ bzgl. der Kriterien Bundesland, Regierungsbezirk und Ortsgröße mit dem Mastersample des Arbeitskreises Deutscher Markt- und Sozialforschungsinstitute e. V. in Bezug gesetzt wurde.

## Erhebungsinstrument

Bei der Entwicklung des Erhebungsinstrumentes galt es, zwei zum Teil widersprüchliche Anforderungen in Einklang zu bringen. Zum einen sollte gewährleistet sein, dass die Trendentwicklung seit 1980 in der Teilstichprobe der Jugendlichen im Alter zwischen 14 und 17 fortgeführt werden kann. Zum anderen machte der Einbezug der Altersgruppe der jungen Erwachsenen zwischen 18 und 25 Jahren im Jahre 2014 eine Anpassung der Fragebogenkonzeption notwendig, da sich deren Lebenssituation vielfach anders darstellt als die der Jugendlichen.

Vor der Erhebung wurde der Fragebogen einem ausführlichen Pretest unter Realbedingungen mit *N* = 40 Jugendlichen und jungen Erwachsenen (quotiert nach Geschlecht, Alter, Bildung und Einwanderungsgeschichte) unterzogen.

Die Interviewdauer betrug im Durchschnitt 41 min. Als Gratifikation für die Teilnahme erhielten sowohl die Jugendlichen und jungen Erwachsenen als auch die Eltern Informationsmaterial der Bundeszentrale für gesundheitliche Aufklärung (BZgA).

## Statistische Analysen

Zur Vorbereitung der Datensätze auf die statistischen Analysen galt es, die disproportionale Stichprobenanlage mithilfe von Designgewichten in eine proportionale zu überführen. Grundlage für die Ermittlung der Gewichte stellten auch hier Veröffentlichungen des Statistischen Bundesamtes dar [11–13]. Auf den Datensatz wurden kombinierte Regional‑, Geschlechter- und Bildungsgewichte sowie für die Gruppe der Befragten mit Migrationshintergrund zusätzlich Gewichte nach Nationalitätsgruppe angewendet. Die Range der Designgewichte beläuft sich auf 0,39 bis 2,72. Alle in diesem Artikel veröffentlichten Ergebnisse werden mit dieser Designgewichtung berichtet.

Deskriptive Analysen geben Aufschluss über das aktuelle Sexual- und Verhütungsverhalten von Jugendlichen und jungen Erwachsenen in Deutschland. Des Weiteren werden in Abhängigkeit vom jeweiligen Skalenniveau zweiseitige χ^2^- bzw. t‑Tests angewendet, um unterschiedliche Verteilungen in Subgruppen bzw. zwischen einzelnen Trendwellen auf Signifikanz zu untersuchen.^3^ Auf Differenzierungen nach soziodemografischen Merkmalen wird weitestgehend verzichtet, da der vorliegende Artikel einen ersten Überblick über erste Ergebnisse der aktuellen Trendwelle bieten soll. Statistische Analysen wurden mit IBM SPSS Version 25 durchgeführt.

Im Falle der Darstellung von Langzeittrends wird auf die Teilstichprobe der Jugendlichen im Alter zwischen 14 und 17 Jahren ohne Migrationshintergrund zurückgegriffen, da für diese Teilstichprobe Trenddaten aus knapp 40 Jahre vorliegen.

## Ergebnisse

### Erleben von Sexualität, Partnerschaft und Liebe

#### Erster Geschlechtsverkehr

**Figure 1:**
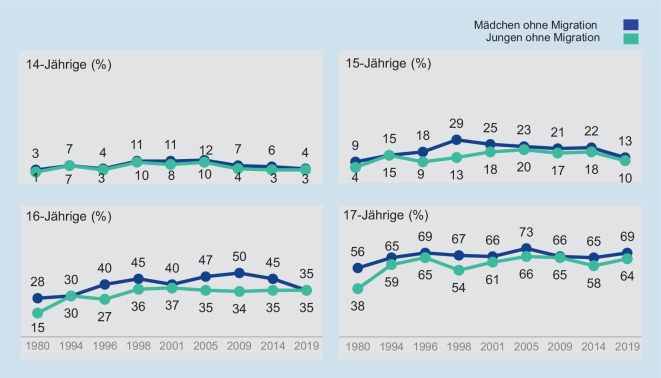

Die Daten der 9. Welle der Jugendsexualitätsstudie zeigen deutlich, dass der Anteil an Jugendlichen mit (heterosexuellen) Geschlechtsverkehrerfahrungen in den letzten Jahrzehnten nicht zugenommen hat (Abb. 1). In der Altersgruppe der 15- und 16-Jährigen ist der Anteil sogar deutlich rückläufig.

Die wenigsten Jugendlichen sind bei ihren ersten Geschlechtsverkehrerfahrungen jünger als 17 Jahre; unter den 16-Jährigen gibt dies beispielsweise nur gut jede bzw. jeder Dritte (34 %) an. Mit steigendem Alter nimmt die sexuelle Aktivität zu: Unter den 17- und 18-Jährigen hat mit jeweils 61 % schon die Mehrheit Geschlechtsverkehr erlebt und ab 22 Jahren berichten rund 9 von 10 jungen Erwachsenen von mindestens einem (heterosexuellen) Geschlechtsakt (88–90 %).

Gefragt nach den Gründen für sexuelle Zurückhaltung^4^ geben die 14- bis 17-Jährigen Jugendlichen an, dass „der oder die Richtige fehle“ (55 %), sie „zu schüchtern seien“ (39 %) und/oder sie sich für „zu jung“ halten (41 %). Auffallend ist in diesem Zusammenhang, dass der Anteil der Mädchen, die sich für „zu jung“ halten, seit 2014 um 13 % zugenommen hat (35 % in 2014 ggü. 48 % in 2019; χ^2^ (1,477) = 8801, *p* = 0,004).

#### Sexuelle Orientierung

9 von 10 Mädchen und jungen Frauen im Alter zwischen 14 und 25 Jahren geben eine heterosexuelle Orientierung an (89 %); bei den Jungen und jungen Männern sind es 93 %.

**Figure 2:**
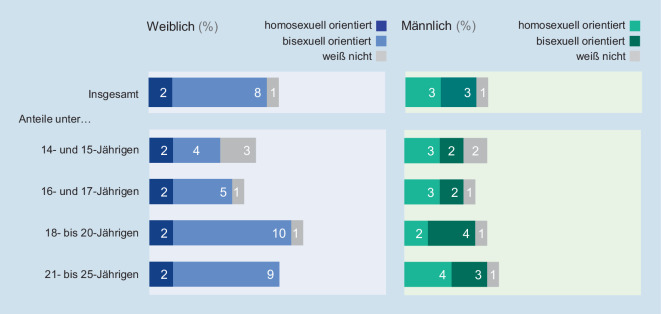

Eine andere als eine rein heterosexuelle Orientierung wird eher von weiblichen als von männlichen Befragten berichtet. Und hier sind es vor allem die jungen Frauen im volljährigen Alter, die am häufigsten angeben, homo- oder bisexuell orientiert zu sein (Abb. 2).

#### Erleben des ersten Geschlechtsverkehrs

Bei den wenigsten Jugendlichen und jungen Erwachsenen läuft der erste Geschlechtsverkehr^5^ spontan ab. Die meisten 14- bis 25-Jährigen geben an, dass es ihnen vor ihrem „ersten Mal“ schon eine Weile klar gewesen sei, dass es „bald“ zum hetero- oder homosexuellen Geschlechtsverkehr kommen würde (47 %). Weitere 31 % wussten, „dass es an diesem Tag passieren würde“, und lediglich gut ein Fünftel (22 %) „hat überhaupt nicht damit gerechnet“. Passend zu diesem Befund, dass für die meisten Befragten der erste Koitus kein zufälliges Ereignis war, erlebte die Mehrheit der Jugendlichen und jungen Erwachsenen den ersten Intimkontakt in einer festen Paarbeziehung (56 %) oder sie waren mit der/dem Sexualpartnerin bzw. -partner gut bekannt (28 %). Ein deutlich geringerer Anteil von 16 % kannte die Person, mit der das „erste Mal“ erlebt wurde, nicht oder nur wenig^6^.

**Figure 3:**
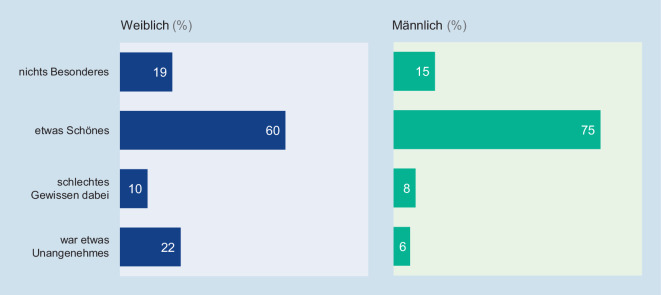

Wie Abb. 3 darstellt, beurteilte die Mehrheit der befragten 14- bis 25-Jährigen den ersten Koitus als „etwas Schönes“. Auffallend ist hier, dass diese Bewertung bei Mädchen und jungen Frauen mit dem Alter zum Zeitpunkt des ersten Geschlechtsverkehrs konfundiert ist: Diejenigen, die angaben beim „ersten Mal“ 14 Jahre oder jünger gewesen zu sein, hatten lediglich zu 44 % ihren ersten Koitus als „etwas Schönes“ erlebt; waren die Befragten beim „ersten Mal“ 15 Jahre oder älter, so bestätigten dies 62 % (χ^2^ (1,1899) = 26.738, *p* = 0,000). Und auch die Bekanntheit mit dem/der Sexualpartner bzw. -partnerin beeinflusst die Bewertung der Mädchen und jungen Frauen: War der Partner bzw. die Partnerin des ersten Intimkontakts den Befragten kaum oder gar nicht bekannt, so gaben lediglich 26 % an, dass dies ein positives Erlebnis gewesen sei. Zum Vergleich: War der Partner bzw. die Partnerin den Befragten gut bekannt oder waren sie fest mit ihm/ihr befreundet, so empfanden 63 % den ersten Koitus als „etwas Schönes“ (χ^2^ (1,1941) = 96.960, *p* = 0,000). Für männliche Befragte können diese Zusammenhänge nicht festgestellt werden.

#### Partnerschaft, Beziehungen und Sexualpartner/-innen

Bezogen auf die Gesamtstichprobe der 14- bis 25-Jährigen geben 41 % der Jugendlichen und jungen Erwachsenen an, sich in einer festen Partnerschaft^7^ zu befinden.

Der Anteil der Jugendlichen und jungen Erwachsenen in festen Partnerschaften variiert mit dem Alter, aber auch mit dem Geschlecht (Tab. 2).

**Table 2:** 

	14–15 Jahre (%)	16–17 Jahre (%)	18–20 Jahre (%)	21–25 Jahre (%)
Mädchen/junge Frauen	7	37	47	66
Jungen/junge Männer	3	11	34	45

Bezüglich der Dauer der Partnerschaften^8^, geben zwei von drei Befragten (65 %) an, dass ihre Partnerschaft bereits seit mindestens einem Jahr bestehe, wobei die Dauer auch wieder mit dem Alter im Zusammenhang steht: Bei den 14- und 15-Jährigen geben 74 % und bei den 16- und 17-Jährigen 63 % an, dass die Partnerschaft erst seit einigen Wochen oder Monaten bestehe. Bei den 18- bis 20-Jährigen beträgt der Anteil nur noch 44 % und in der ältesten Kohorte der 21- bis 25-Jährigen geht er auf 23 % zurück. 18- bis 20-Jährige leben nach eigenen Angaben zu 57 % bereits länger als ein Jahr in einer festen Beziehung.

Wenn also volljährige, junge Erwachsene in einer Paarbeziehung leben, so dauern diese in der Mehrheit der Fälle länger an. Und in diesen Beziehungen ist sexuelle Treue^9^ für viele junge Menschen von großer Bedeutung – für die jungen Frauen mehr als für die jungen Männer. 75 % der weiblichen und 62 % der männlichen Befragten zwischen 18 und 25 Jahren halten sexuelle Treue für „unbedingt notwendig“ (χ^2^ (1,4171) = 79.086, *p* = 0,000) und nur wenige finden die Forderung nach sexueller Treue „falsch“ (3 % der weiblichen ggü. 6 % der männlichen Befragten; χ^2^ (1,4170) = 25.935, *p* = 0,000); weitere 32 % der jungen Männer und 22 % der jungen Frauen geben sexuelle Treue als „wünschenswert“ an (χ^2^ (1,4170) = 44.318, *p* = 0,000).

Das Erleben von Sexualität scheint somit normativ für viele junge Erwachsene an eine Partnerschaft gebunden zu sein. Zu diesem Befund passt auch, dass junge Erwachsene, die zum Zeitpunkt der Befragung in einer Partnerschaft leben, deutlich häufiger „regelmäßigen“ Geschlechtsverkehr angegeben als Befragte ohne Partnerschaft (85 % ggü. 18 % ohne Partnerschaft).^10^

**Figure 4:**
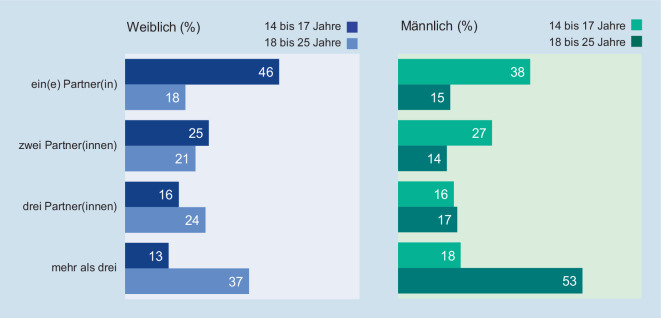

Mit steigendem Alter nimmt auch die Anzahl der bisherigen Sexualpartnerinnen und -partner zu. Wie Abb. 4 zeigt, hat die deutliche Mehrzahl der Jugendlichen zwischen 14 und 17 Jahren bis dahin lediglich ein oder zwei Sexualpartnerinnen oder -partner gehabt. Mit zunehmendem Alter nimmt die Gesamtzahl der bisherigen Sexualpartnerinnen und -partner zu.

### Kontrazeptionsverhalten

#### Nichtverhütung beim ersten Geschlechtsverkehr

Um zuverlässige Informationen über die Verhütungsquote bei jungen Menschen in Deutschland zu erhalten, wird die Zielgruppe der 14- bis 17-Jährigen fokussiert, da bei Minderjährigen ein Kinderwunsch i. d. R. ausgeschlossen werden kann (lediglich 1 % der Jugendlichen würden in der vorliegenden Befragung eine aktuelle Schwangerschaft für „erfreulich“ halten).

**Figure 5:**
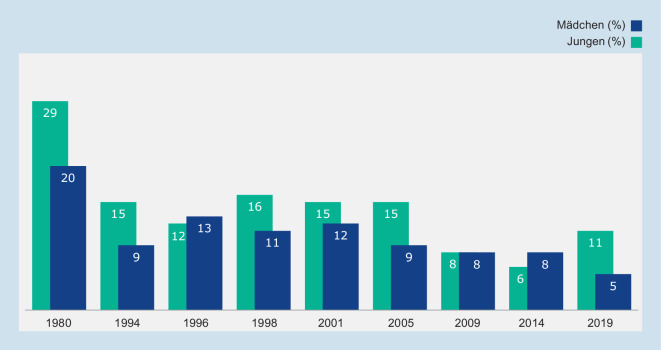

Insgesamt geben 9 % der 14- bis 17-Jährigen an, beim ersten Sexualverkehr nicht verhütet zu haben. Abb. 5 stellt die Trendentwicklung der vergangenen knapp 40 Jahre für Jugendliche ohne Migrationshintergrund dar. Trotz des deutlich positiven Trends zeigt sich hier ein leicht ansteigender Anteil an männlichen Jugendlichen, der nicht verhütet, wenngleich diese Veränderung nicht signifikant ist (6 % in 2014 ggü. 11 % in 2019; χ^2^ (1,380) = 2,277, *p* = 0,143).

Als Gründe für ausbleibende Verhütung^11^ geben Jugendliche am häufigsten an, dass „es zu spontan kam“ (56 %), dass sie annahmen, „es werde schon nichts passieren“ (38 %), und dass „so spontan keine Verhütungsmittel griffbereit waren“ (27 %).

#### Wahl der Verhütungsmittel

Jugendliche verfügen über ein breites Wissen zu Verhütung und Sexualität, wie die geringe Quote der Nichtverhütenden beim ersten Geschlechtsverkehr zeigt. Die wichtigsten Quellen der Aufklärung über Verhütungsmittel^12^ für 14- bis 17-Jährige sind der Schulunterricht (69 %), Gespräche (68 %) und das Internet (59 %). Und auch Jugendzeitschriften werden von den Jugendlichen nach eigenen Angaben häufig zur Wissensgewinnung herangezogen (34 %).

Die Daten der 9. Welle der Jugendsexualitätsstudie zeigen, dass sich die Wahl des Verhütungsmittels mit dem Alter, zunehmender sexueller Erfahrung sowie dem Vorhandensein einer Partnerschaft verändert.

**Figure 6:**
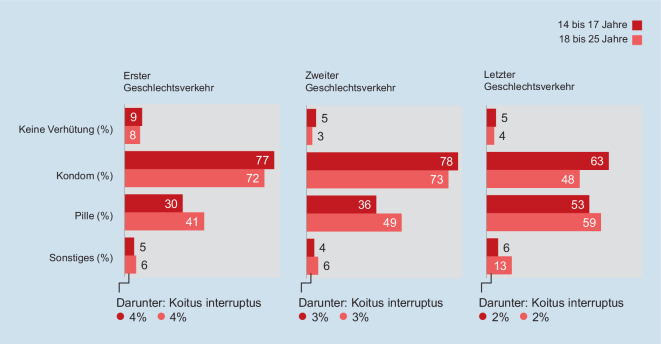

Bei den ersten Geschlechtsverkehrerfahrungen geben sowohl Jugendliche als auch junge Erwachsene deutlich häufiger an, mit dem Kondom als mit der Pille verhütet zu haben (Abb. 6). Beim letzten Geschlechtsverkehr hingegen reduziert sich der Anteil der Befragten, die ein Kondom eingesetzt haben, und die Pille gewinnt an Bedeutung. Und gerade in Partnerschaften verhüten die Befragten häufiger mit der Pille (65 % ggü. 49 % Pillennutzung ohne aktuelle Partnerschaft; χ^2^ (1,3874) = 90.581, *p* = 0,000).

Eine nur untergeordnete Rolle spielen andere Verhütungsmittel, wie beispielsweise (Kupfer‑)Spirale oder das Diaphragma (Anteil der Nutzerinnen 0–4 %).

#### Im Fokus: Die Pille

Obwohl Mädchen und junge Frauen gerade in Partnerschaften die Pille häufig zur Kontrazeption einsetzten, ist der Anteil der Nutzung im Trendvergleich seit 2014 rückläufig. Der Rückgang in der Nutzungshäufigkeit beläuft sich unabhängig vom Vorhandensein einer Partnerschaft auf 13 bzw. 12 Prozentpunkte (Verhütung beim letzten Geschlechtsverkehr: Befragte in Partnerschaft: 78 % in 2014 ggü. 65 % in 2019 (χ^2^ (1,2650) = 50.104, *p* = 0,000); Befragte außerhalb Partnerschaft: 66 % in 2014 ggü. 54 % in 2019 (χ^2^ (1,1295) = 18.107, *p* = 0,000)).

**Figure 7:**
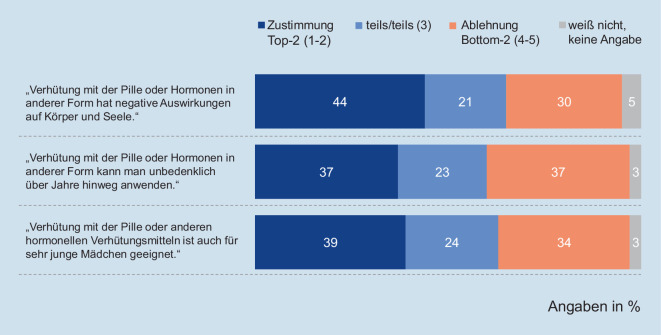

Mit dem deutlichen Rückgang der Pillennutzung geht zudem eine insgesamt skeptischere Haltung gegenüber der Pille einher: Die Gesundheitsverträglichkeit^13^ wurde 2019 schlechter als noch 2014 bewertet (2014: MD = 2,64, SD = 1,35; 2019: MD = 3,08, SD = 1,51; t (3650) = −9362, *p* = 0,000). Und auch die Einstellung von Mädchen und jungen Frauen gegenüber hormoneller Verhütung ist insgesamt uneinheitlich und tendenziell eher kritisch bzw. ambivalent (Abb. 7).

#### Notfallkontrazeption

Das Wissen der 14- bis 25-jährigen sexuell aktiven Mädchen und jungen Frauen um die Möglichkeit einer Notfallverhütung durch die „Pille danach“ ist nahezu flächendeckend vorhanden: 96 % geben an, diese Notfallkontrazeption zu kennen.^14^

Gut ein Viertel (27 %) der Befragten hat nach eigenen Angaben mit der „Pille danach“ schon einmal eine Notfallverhütung vorgenommen, 9 % auch schon mehrfach.^15^

Die Anwendungshäufigkeit in der Stichprobe der 14- bis 25-Jährigen steht mit dem Alter im Zusammenhang: Unter den minderjährigen sexuell aktiven Mädchen ist die Nutzung einer Notfallkontrazeption nur halb so verbreitet (einmal: 10 %; mehrmals: 5 %) wie unter den volljährigen jungen Frauen (einmal: 20 %; mehrmals: 9 %). Je älter die Frauen und je länger sie sexuell aktiv sind, desto wahrscheinlicher ist es, dass sie die „Pille danach“ schon einmal angewendet haben.

Anhand der Daten der Jugendsexualitätsstudie kann eine Langzeitentwicklung der Nutzung einer Notfallkontrazeption durch Mädchen zwischen 14 und 17 Jahren ohne Migrationshintergrund aufgezeigt werden: Die einmalige Nutzung hat sich nicht bedeutsam verändert (8 % in 2001 ggü. 10 % in 2019). Die Mehrfachnutzung ist von 1 % in 2001 auf 4 % in 2019 angestiegen.

## Diskussion

Die 9. Welle der Jugendsexualitätsstudie liefert auf Basis repräsentativer Stichproben Informationen über das Sexual- und Verhütungsverhalten der aktuellen Generation der 14- bis 25-Jährigen in Deutschland.

Vor allem das „erste Mal“ ist ein gesellschaftlich viel diskutiertes Thema. Die vorliegenden Daten liefern keine Hinweise darauf, dass Jugendliche bei ihren ersten Koituserfahrungen immer jünger werden. Vielmehr setzt sich in Deutschland der Trend, dass Jugendliche immer später sexuell aktiv werden, auch 2019 fort [7]. Hier liegt eine große Stärke der repräsentativen Jugendsexualitätsstudie: Der Einstieg ins aktive Sexualleben wird nicht retrospektiv von den Befragten angegeben, sondern die jeweils aktuelle Generation wird zu vorhandenen sexuellen Erfahrungen befragt. Damit können Verzerrungen im Antwortverhalten aufgrund von Erinnerungseffekten ausgeschlossen werden.

Die meisten Jugendlichen und jungen Erwachsenen erleben den ersten homo- oder heterosexuellen Geschlechtsverkehr in einer festen Partnerschaft und als ein geplantes Ereignis. Das „erste Mal“ wird auch überwiegend als „etwas Schönes“ bewertet. Diese Bewertung fällt bei Mädchen und jungen Frauen jedoch deutlich seltener positiv aus, wenn sie beim ersten Koitus sehr jung waren oder ihnen die Person, mit der sie das „erste Mal“ erlebten, nur wenig oder gar nicht bekannt war.

Insgesamt findet somit das positive Erleben von Sexualität für Jugendliche und junge Erwachsene in der Regel in Paarbeziehungen statt. Der häufige Wechsel von Sexualpartnerinnen und -partnern ist gerade bei Minderjährigen die Ausnahme. Und die Mehrheit der jungen Erwachsenen – junge Frauen häufiger als junge Männer – wünscht sich monogame Beziehungen bzw. hält sexuelle Treue sogar für unverzichtbar.

Zur sexuellen Orientierung von jungen Menschen in Deutschland können im Rahmen der Jugendsexualitätsstudie erstmals repräsentative Informationen vorgelegt werden. 11 % der weiblichen und 7 % der männlichen Jugendlichen und jungen Erwachsenen geben eine bi- oder homosexuelle Orientierung an. Dieser Anteil ist damit höher als bei Erwachsenen [14].

Im Hinblick auf die Kontrazeption zeigen die aktuellen Daten, dass die meisten Jugendlichen in Deutschland sicher verhüten. Aktuell geben 5 % an, beim letzten Geschlechtsverkehr nicht verhütet zu haben. Diese Quote ist im Vergleich zum Durchschnitt von 30 europäischen und außereuropäischen Industrienationen sehr niedrig [15].

Die Wahl des Verhütungsmittels ist mit dem Alter und dem damit einhergehenden Ausmaß an sexueller Erfahrung bzw. dem Vorhandensein einer Partnerschaft konfundiert. In jungen Jahren und mit wenigen sexuellen Erfahrungen verhüten Jugendliche vor allem mit dem Kondom, seltener mit der Pille. Mit zunehmendem Alter und dem Vorhandensein einer länger andauernden Paarbeziehung nimmt die Nutzungshäufigkeit der Pille deutlich zu. Dieses Nutzungsmuster ist auch in einer kanadischen Studie beschrieben [16].

Aber obwohl viele junge Menschen gerade in Partnerschaften häufig mit der Pille verhüten, zeigen sich in den vorliegenden Daten Hinweise auf einen möglichen Einstellungswandel gegenüber hormoneller Verhütung: Der Anteil der Befragten, der die Pille zur Verhütung einsetzt, ist rückläufig. Diese Entwicklung deckt sich mit dem Rückgang der Pillenverordnung bei gesetzlich versicherten Mädchen und jungen Frauen [17]. Doch nicht nur die Anwendungshäufigkeit der Pille ist rückläufig, auch die Gesundheitsverträglichkeit wird schlechter bewertet, wie die aktuellen Daten der Jugendsexualitätsstudie belegen. Dass diese skeptische Einstellung auch mit allgemein gewandelten Normvorstellungen im Zusammenhang stehen könnte, legen andere Jugendsurveys nahe: Die aktuelle Shell-Jugendstudie beispielsweise belegt, dass eine bewusste Lebensführung mit einem hohen Gesundheitsbewusstsein für Jugendliche heute wichtiger ist als noch vor einigen Jahren [18]. Und gesundheitsbezogene Aspekte spielen bei der Wahl der Verhütungsmethode für Mädchen eine relevante Rolle, wie eine Studie aus den USA belegte [19]. Damit könnte eine hormonfreie Verhütung als erstrebenswertes Verhalten im Kontext eines ökologisch nachhaltigen und gesundheitsbewussten Lebensstils angesehen werden. Diese Einstellung und daraus abgeleitete Lebensweisen werden u. a. auch durch soziale Medien vermittelt, in denen beispielsweise Influencerinnen mit großen Reichweiten eine hormonfreie Verhütung als erstrebenswertes Ideal vorleben [20].

Abschließend zeigt sich im Hinblick auf eine Notfallkontrazeption, dass das Wissen der 14- bis 25-jährigen Mädchen und jungen Frauen um diese Möglichkeit fast flächendeckend vorhanden und die einmalige Nutzung unter Jugendlichen seit fast 20 Jahren überwiegend konstant ist. In Bezug auf die mehrmalige Nutzung zeigt sich ein Anstieg bei einer insgesamt sehr geringen Anwendungshäufigkeit.

Insgesamt zeigen die aktuellen Ergebnisse der Jugendsexualitätsstudie in Bezug auf die Kontrazeption, dass Jugendliche und junge Erwachsene in Deutschland über ein breites Wissen über Sexualität und Familienplanung verfügen, was sich in einer hohen Verhütungskompetenz niederschlägt. Dies zeigt sich auch in dem kontinuierlichen Rückgang der Schwangerschaften und Schwangerschaftsabbrüche bei Mädchen unter 18 Jahren [21, 22]. Daraus kann geschlussfolgert werden, dass Informationen zur Aufklärung die jungen Menschen in Deutschland erreichen und sie diese auch in Anspruch nehmen. Wichtige Instanzen der Wissensvermittlung sind hier nach wie vor die Schulen, Gespräche in den Elternhäusern, aber auch der Austausch unter Gleichaltrigen und das Internet [23]. Aufklärungsmaßnahmen leisten somit einen bedeutsamen Beitrag zur sexuellen Gesundheitsförderung von jungen Menschen und beugen präventiv Schwangerschaftskonflikten vor.

Abschließend sei erwähnt, dass die vorliegende Trendwelle der Jugendsexualitätsstudie im Jahre 2019 – also vor Ausbruch der COVID-19-Pandemie – durchgeführt wurde. Erkenntnisse über die Auswirkungen der kontaktbeschränkenden Maßnahmen auf das Sexual- und Verhütungsverhalten von Jugendlichen und jungen Erwachsenen können somit aus den vorliegenden Daten nicht abgeleitet werden. Angebote der sexuellen Bildung und Prävention jedoch mussten aufgrund der COVID-19-Pandemie nahezu komplett eingestellt werden [24, 25]. Die Auswirkungen dieser Entwicklung auf die sexuelle und reproduktive Gesundheit von jungen Menschen in Deutschland werden voraussichtlich in der nächsten Trendwelle sichtbar sein, die sich derzeit in Planung befindet.

## Fazit

Zusammenfassend belegen die Daten der 9. Welle der Jugendsexualitätsstudie ein weitgehend sicheres Kontrazeptionsverhalten von jungen Menschen in Deutschland. Im Bereich hormoneller Verhütung scheint sich ein Einstellungswandel in der kommenden Generation bemerkbar zu machen.

Es gilt das Engagement im Bereich Sexualaufklärung und Familienplanung aufrechtzuerhalten bzw. zu intensivieren. Nur so können die sexuelle und reproduktive Gesundheit der nachfolgenden Generationen gewährleistet und mögliche negative Folgen der COVID-19-Pandemie reduziert werden.

## Footnotes

1 Aufgrund der methodischen Anlage der Jugendsexualitätsstudie muss auf eine weitere nonbinäre Ausdifferenzierung des Geschlechts verzichtet werden. Die Autorenschaft möchte an dieser Stelle explizit darauf hinweisen, dass dieses Vorgehen alleine auf methodischen Notwendigkeiten und nicht auf eine nicht vorhandene diversitätssensible Perspektive zurückzuführen ist.

2 Stichtag 31.12.2017.

3 Der Anteil an fehlenden Werten pro Item (Item No Response) ist aufgrund der Face-to-Face-Situation und des Selbstausfüllerteils gering. Über alle Items des Fragebogens beläuft sich deren Anteil im Maximum auf 16 % (Item: Erfahrung mit verschiedenen Verhütungsmitteln, HIER: Diaphragma, Pessar, Portiokappe). Fehlende Werte wurden der Restkategorie („weiß nicht“) zugeordnet. Unvollständige Datensätze (Unit No Response) sind nicht vorhanden.

4 Fragestellung: „Markieren Sie bitte die Gründe, die für Sie entscheidend waren, keinen engeren Kontakt einzugehen.“ (Mehrfachnennungen möglich); Basis: 14- bis 17-Jährige ohne bisherigen Körperkontakt (*n* *=* 906, ungewichtet).

5 Fragestellung: „Welche der folgenden Feststellungen kennzeichnet Ihre Situation vor dem ersten Geschlechtsverkehr bzw. Sex am besten?“; Basis: 14- bis 25-Jährige mit heterosexueller Geschlechtsverkehrerfahrung/homosexuellen Intimkontakten (*n* *=* 3078, ungewichtet).

6 Fragestellung: „Wie genau kannten Sie den Partner, mit dem, bzw. die Partnerin, mit der Sie zum ersten Mal Geschlechtsverkehr bzw. Sex hatten?“; Basis: 14- bis 25-Jährige mit heterosexueller Geschlechtsverkehrerfahrung/homosexuellen Intimkontakten (*n* *=* 3078, ungewichtet).

7 Fragestellung: „Haben Sie derzeit eine feste Beziehung?“; Basis: 14- bis 25-Jährige (*N* = 6032, ungewichtet).

8 Fragestellung: „Wie lange sind Sie mit Ihrem jetzigen Partner/Ihrer jetzigen Partnerin schon fest zusammen?“; Basis: 14- bis 25-Jährige in fester Partnerschaft bzw. verheiratet, zusammenlebend oder in eingetragener Partnerschaft (*n* = 2049, ungewichtet).

9 Fragestellung: „Wie ist Ihre Einstellung zu sexueller Treue in einer Partnerschaft?“; Basis: 18- bis 25-Jährige (*n* = 2476, ungewichtet), Frage wurde nur der Teilstichprobe der 18- bis 25-Jährigen gestellt.

10 Fragestellung: „Haben Sie derzeit nur zeitweise/gelegentlich Geschlechtsverkehr oder regelmäßig?“; Basis: 18- bis 25-Jährige mit mehrmaliger heterosexueller Geschlechtsverkehrerfahrung (*n* = 1998, ungewichtet).

11 Fragestellung: „Was waren die Gründe dafür, dass Sie bei Ihrem ersten Geschlechtsverkehr nicht verhütet haben?“ (Mehrfachnennungen möglich); Basis: 14- bis 25-Jährige mit heterosexueller Geschlechtsverkehrerfahrung und ohne Verhütung beim ersten Mal (*n* *=* 241, ungewichtet).

12 Fragestellung: „Woher stammen Ihre Kenntnisse über Sexualität, Fortpflanzung, Empfängnisverhütung usw. überwiegend?“ (Mehrfachnennungen möglich); Basis: 14- bis 17-Jährige (*n* *=* 3556, ungewichtet).

13 Fragestellung: „Im Folgenden wollen wir von Ihnen wissen, wie bestimmte Eigenschaften von Verhütungsmitteln von Ihnen eingeschätzt werden … Bewerten Sie bitte jetzt die Antibabypille.“; Skala von 1 „sehr gut“ bis 6 „sehr schlecht“; Basis: 14- bis 25-jährige Mädchen/Frauen mit mehrmaliger heterosexueller Geschlechtsverkehrerfahrung (*n* *=* 1837, ungewichtet).

14 Fragestellung: „Nach einer Verhütungspanne oder nach einem ungeschützten Geschlechtsverkehr gibt es die Möglichkeit der Notfallverhütung, die sogenannte ‚Pille danach‘. [Nähere Erläuterungen] Wussten Sie, dass es so eine Pille gibt?“; Basis: 14- bis 25-jährige Mädchen/junge Frauen mit heterosexueller Geschlechtsverkehrerfahrung (*n* *=* 1886, ungewichtet).

15 Fragestellung: „[Nähere Erläuterung] Haben Sie die ‚Pille danach‘ selbst schon angewendet?“; Basis: 14- bis 25-jährige Mädchen/Frauen mit mehrmaliger heterosexueller Geschlechtsverkehrerfahrung (*n* *=* 1837, ungewichtet).
